# High affinity complexes of pannexin channels and L-type calcium channel splice-variants in human lung: Possible role in clevidipine-induced dyspnea relief in acute heart failure

**DOI:** 10.1016/j.ebiom.2016.06.027

**Published:** 2016-06-22

**Authors:** Gerhard P. Dahl, Gregory E. Conner, Feng Qiu, Junjie Wang, Edward Spindler, Jason A. Campagna, H. Peter Larsson

**Affiliations:** a Department of Physiology and Biophysics, University of Miami, Miller School of Medicine, Miami, Florida, United States; b Department of Cell Biology, University of Miami, Miller School of Medicine, Miami Florida, United States; c Surgery and Perioperative Care, The Medicines Company, Parsippany, NJ, United States

**Keywords:** Clevidipine, Voltage-gated calcium channels, CaV1.2, Pannexin, Dyspnea, Hypertensive heart failure

## Abstract

Clevidipine, a dihydropyridine (DHP) analogue, lowers blood pressure (BP) by inhibiting l-type calcium channels (Ca_V_1.2; gene *CACNA1C*) predominantly located in vascular smooth muscle (VSM). However, clinical observations suggest that clevidipine acts by a more complex mechanism. Clevidipine more potently reduces pulmonary vascular resistance (PVR) than systemic vascular resistance and its spectrum of effects on PVR are not shared by other DHPs. Clevidipine has potent spasmolytic effects in peripheral arteries at doses that are sub-clinical for BP lowering and, in hypertensive acute heart failure, clevidipine, but not other DHPs, provides dyspnea relief, partially independent of BP reduction. These observations suggest that a molecular variation in Ca_V_1.2 may exist which confers unique pharmacology to different DHPs. We sequenced *CACNA1C* transcripts from human lungs and measured their affinity for clevidipine. Human lung tissue contains *CACNA1C* mRNA with many different splice variations. Ca_V_1.2 channels with a specific combination of variable exons showed higher affinity for clevidipine, well below the concentration associated with BP reduction. Co-expression with pannexin 1 further increased the clevidipine affinity for this Ca_V_1.2 splice variant. A high-affinity splice variant of Ca_V_1.2 in combination with pannexin 1 could underlie the selective effects of clevidipine on pulmonary arterial pressure and on dyspnea.

**Research in Context:**

Clevidipine lowers blood pressure by inhibiting calcium channels in vascular smooth muscle. In patients with acute heart failure, clevidipine was shown to relieve breathing problems. This was only partially related to the blood pressure lowering actions of clevidipine and not conferred by another calcium channel inhibitor. We here found calcium channel variants in human lung that are more selectively inhibited by clevidipine, especially when associated with pannexin channels. This study gives a possible mechanism for clevidipine's relief of breathing problems and supports future clinical trials testing the role of clevidipine in the treatment of acute heart failure.

## Highlights

• CaV1.2 splice variants are found in human lung that have increased affinity for clevidipine.
• Co-expression of CaV1.2 splice variant with Pannexin 1 further increases affinity for clevidipine but not for nicardipine.
• Study supports future clinical trials testing the role of clevidipine in the treatment of acute hypertensive heart failure.

## 1 Introduction

**Fig. 1:**
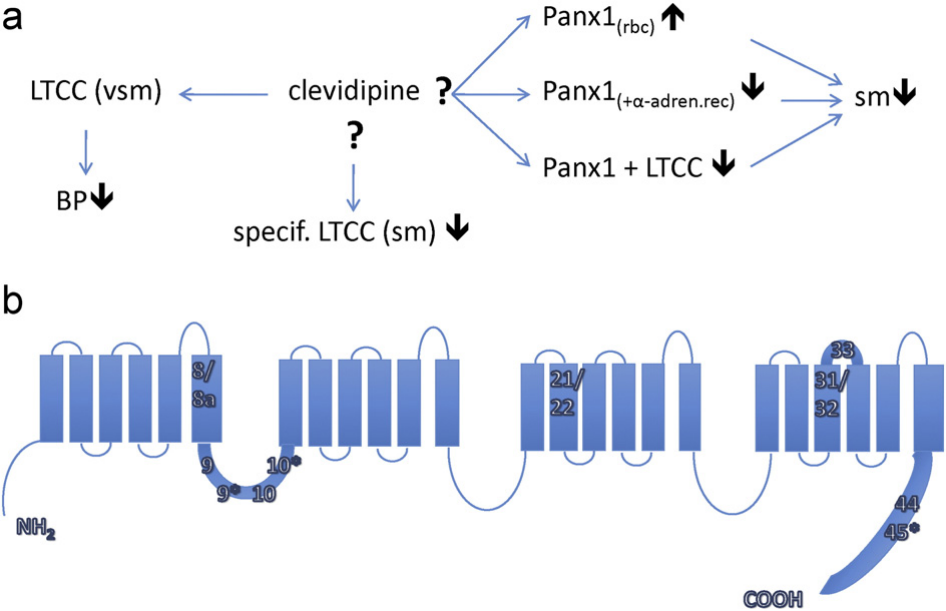
Putative Clevidipine targets for lowering blood pressure. A) Scheme showing putative Clevidipine targets (including Panx1) to lower blood pressure in lung and other tissues. B) Ca_V_1.2 α-1 subunit topology and location of spliced exons examined in this study.

Clevidipine is a 3rd generation dihydropyridine (DHP) that lowers blood pressure via selective antagonism of peripheral vascular smooth muscle (VSM) voltage-gated l-type calcium channels (Ca_V_1.2) (Fig. 1A). Recently, clinical observations suggest that the mechanisms of clevidipine action are more complex than simple antagonism of peripheral VSM Ca_V_1.2. In a clinical trial examining the safety and efficacy of clevidipine for blood pressure (BP) reduction in hypertensive acute heart failure (AHF), clevidipine showed a superior dyspnea-relieving benefit versus standard of care intravenous (IV) antihypertensives, including another DHP (Peacock et al., 2014). When compared to standard of care, dyspnea relief in patients receiving clevidipine was more robust and faster, and this benefit was only partially related to the blood pressure lowering actions of clevidipine. Importantly, this benefit was not conferred by nicardipine, another l-type blocking DHP agent. Clevidipine is also a potent pulmonary vasodilator with most of the PVR reduction occurring at doses that are lower than those needed to cause the majority of the reduction of systemic blood pressure (Cleviprex package insert and (Kieler-Jensen et al., 2000)) (Fig. 1A). Clevidipine effects on PVR as measured by overall response rate and magnitude of reduction are equivalent to the nitrovasodilators and clevidipine is also effective in nitrate non-responders (Nordlander et al., 2004). Lastly, similar to the observed potency increase for clevidipine effects on pulmonary vascular resistance (PVR), clevidipine has a spasmolytic effect in peripheral arteries that occurs at doses that are minimally effective for BP reduction (Huraux et al., 1997, Patel et al., 2012). This dose-response relationship between spasmolysis and BP reduction is not shared by other DHPs (Schmidt et al., 2010). In this study, we applied a reverse translational approach to understand these non-BP lowering effects of clevidipine by conducting further basic science studies on an already approved clinical drug.

In the nearly 18 years since the clevidipine investigational new drug application was filed, our understanding of the structure, tissue distribution and molecular biology of the l-type calcium channels (LTCC) has evolved dramatically (Zuccotti et al., 2011, Abernethy and Soldatov, 2002). The Ca_V_-α-1 family comprises 10 genes, of which 4 genes (*CACNA1S*, *CACNA1C*, *CACNA1D CACNA1F*) encode the LTCC referred to as Ca_V_1.1, Ca_V_1.2, Ca_V_1.3 and Ca_V_1.4 channels. LTCC are distinguished from the other 6 Ca_V_ channels by their selective sensitivity to 1,4-dihydropyridines (DHPs), phenylalkylamines (PAAs) and benzothiazepines and by their characteristic slowly inactivating currents. LTCCs are composed of a central pore-forming α-1 subunit and additional α-2/δ, β, and γ subunits. The α-1 subunit confers most of the functional properties to the channel, including voltage sensing, permeability, calcium-dependent inactivation, and sensitivity to organic channel blockers. Although the α-1 subunit defines the basic channel properties, four different β genes (β1–β4; genes *CACNB1–4*) and extensive splice variants of each gene exist that distinctly modify activation and inactivation kinetics, voltage gating, and drug sensitivity (Hullin et al., 2003).

At least 20 of the 56 exons in the human *CACNA1C* transcript are alternatively spliced (Liao et al., 2007) (Fig. 1B). Splice variants are known to confer different electrophysiological and pharmacological properties on the Ca_V_1.2 channel and to exhibit tissue-specific differences (e.g. cardiac muscle vs. vascular smooth muscle (VSM)) (Liao et al., 2007, Cheng et al., 2009). Smooth muscle is known to be more sensitive to DHPs than cardiac muscle (Moosmang et al., 2003). These tissues express slightly different Ca_V_1.2 splice variants (Cheng et al., 2009, Saada et al., 2003, Liao et al., 2005): exon 8 is expressed in smooth muscle, while exon 8a is expressed in cardiac muscle. Exon 8a in cardiac tissue reduces the affinity of Ca_V_1.2 for DHPs (Welling et al., 1997). Lastly, in addition to the molecular heterogeneity conferred by differing subunit combinations and alternative splice variants, disease-based differences in tissue distribution and expression levels of any given channel complex are common (Hullin et al., 2003, Firth et al., 2011). In this report, we tested the hypothesis that, in lung tissue, there are specific *CACNA1C* splice variants encoding for Ca_V_1.2 with different molecular pharmacologic profiles for clevidipine compared to Ca_V_1.2 in other peripheral smooth muscles (Fig. 1A).

In parallel to our increased understanding of Ca_V_1.2 channels during the last decade, a more detailed understanding of the molecular basis of BP regulation has emerged. For example, pannexin 1 (gene *PANX1*), which serves as the major ATP-release channel in many cell types (including erythrocytes, endothelial cells, airway epithelial cells and astrocytes (Locovei et al., 2006a, Ransford et al., 2009, Dahl and Keane, 2012)), is involved in two antagonistic ways for blood flow and blood pressure regulation. 1) Erythrocytes sensing low oxygen content and/or subjected to shear stress release ATP through Panx1 channels (Locovei et al., 2006a, Sridharan et al., 2010). The ATP binds to purinergic receptors on endothelial cells, triggering a propagated calcium wave that eventually results in the release of nitric oxide (NO). NO then relaxes vascular smooth muscle cells which increases local perfusion and oxygen supply (Fig. 1A). 2) Activation of α-adrenergic receptors leads to opening of Panx1 channels in VSM cells and blocking of Panx1 channels in these cells attenuates the vasopressor activity of α-agonists (Billaud et al., 2012). Thus, it appears that Panx1 is tied into the α-adrenergic control of blood pressure (Fig. 1A). In this report, we therefore also considered the hypotheses that the clevidipine-induced dyspnea relief is due to clevidipine acting on Panx1 in lung tissue. It is also known that Panx1 co-localizes with voltage-gated calcium channels (Ca_V_1.1) in skeletal muscle (Jorquera et al., 2013). We therefore also considered the possibility that Panx1 associates with Ca_V_1.2 in lung tissue and increases the affinity of Ca_V_1.2 to clevidipine.

## 2 Methods

### 2.1 Identification of Ca_V_1.2 Splice Variants in Lung Tissue

Human lung tissue (8 donors) without overt disease, but not suitable for transplant, was obtained from the Life Alliance Organ Recovery Agency according to institutional review board guidelines regarding consent and de-identification of individual donors (demographic characteristics, see Table 1). Peripheral lung tissue was excised, chopped into ~ 5 mm fragments, and snap frozen in liquid N_2_. For preparation of total RNA, tissue was ground in a mortar and pestle under liquid N_2_ and the powder immediately extracted using E.Z.N.A. HP Total RNA Isolation Kit (Omega Bio-Tek, Norcross, GA). Total RNA was treated with DNase I and reverse transcribed using AMV First Strand cDNA Synthesis Kit (New England Biolabs, Ipswich, MA) with a primer specific in exon 50 of the *CACNA1C* transcript 3′ UTR (see Table 1 for primers sequences). cDNA was amplified by 35 cycles of PCR using Hotstar Hifidelity Taq polymerase kit (Qiagen, Valencia, CA) with primers specific for exons surrounding known spice sites (Table 2) and the PCR reactions cloned into pGEMTeasy. cDNA in random individual colonies was isolated and sequenced.

### 2.2 Electrophysiology

Eight different full-length *CACNA1C* cDNAs (Table 3) were constructed by combining different exons from the Ca_V_1.2 splice variants found in the human lung. These eight cDNAs were transferred into a Xenopus oocyte expression vector and mRNAs were transcribed in vitro. 50 nl of 1 μg/μl mRNA of the different splice variants of the α-1 subunit of Ca_V_1.2 together with its α-2/δ and β subunits were injected into Xenopus oocytes. Currents were recorded 2–5 days after mRNA injection using two-electrode voltage clamp technique. Currents were filtered at 500 Hz and sampled at 5 kHz. Extracellular solution contained (in mM): 20 barium acetate, 70 sodium glutamate, 5 HEPES, 2 KOH, pH = 7.3. For recordings with Panx1, in vitro transcribed mRNAs for Panx1 together with mRNA for alpha-1, alpha-2/delta and beta subunits of Ca_V_1.2 were injected into Xenopus oocytes at equal ratios. Results are given as mean ± SEM.

### 2.3 Funding

This study was funded by The Medicines Company (Parsippany NJ) and a University of Miami SAC Award 2016-31R to GEC and HPL. Funders had no role in study conduct, experimental conduct, data collection or data analysis.

## 3 Results

### 3.1 Many Different Ca_V_1.2 Splice Variants Present in Lung Tissue From Individuals

Because clevidipine has greater potency for reducing PVR, it is possible that, in human lungs, there is expressed a unique combination of Ca_V_1.2 splice variants with a higher affinity for clevidipine that Ca_V_1.2 expressed in other tissues. To test this idea, cDNA was prepared from human lung parenchyma and used to amplify regions of *CACNA1C* known to have substantial splicing variation: exons 7–11 (7,8,8a,9,9*,10,10*,11), 20–24 (20,21,22,23,24), 30–35 (30,31,32,33,34) and 40–46 (40,41,42,43,44,45,46) (Fig. 1). Amplimers were not seen in controls using RNA without reverse transcription and 35 cycles of amplification. Interestingly, different individuals displayed different frequencies of individual splice variants (Table 2).

Five different variants were detected between exons 7 and 11. As expected, only exon 8, and not the cardiac-specific alternate exon 8a, was seen (Welling et al., 1997). The most common variant contained Exons 8, 9 and 10, skipped exons 9* and 10* (7,8,-,9,-,10,-,11), and was present in all individuals examined. This variant represented 74% of all amplified cDNA fragments in this region (Table 2). The variant with exons 8, 9, 9* and 10 (7,8,-,9,9*,10,-,11) was seen in 4 of 6 individuals and varied between 10 and 40% of the total isolates from an individual (Table 2). Exons 8, 9, 10 and 10* (7,8,-,9,-,10,10*,11) and exons 8, 9, 9*, 10, 10* (7,8,9,9*,10,10*,11) were seen in two of six individuals. Another variant was detected that deleted exons 8, 9, 9* and 10* (7,-,-,-10,-11). Exon 8 is believed to be required for functional Ca_V_1.2 expression and thus this variant is mostly likely not functional.

Only two splice variants were detected between exons 20 and 24 (20,21,-,23,24 and 20,-,22,23,24). Previously reported alternate exons 21 and 22 were never both present in an individual (Table 2). Exon 21 was present in seven of eight individuals.

Significant variation was apparent between exons 30 and 34. Ten different splice variants were detected, four of which were not expected to be functional (Table 2). Two variants differing by the alternate exons 31 and 32 in combination with exon 33 (30,31,-,33,34 or 30,-,32,33,34) were present in all individuals (*n* = 7) examined, with exon 32 ranging from 20 to 50% of clones from a single individual and alternate exon 31 ranging between 10 and 25% in a single individual. In addition, exon 32 in the absence of exon 33(Δ33) was present in six of seven individuals (30,-,32,-,34) with a frequency of 15–30%. Exon 31 in the absence of exon 33 (30,31,-,-,34) was seen with less frequency (Table 2).

Only two splice variants between exons 40 and 46 were detected, both lacking exon 45 (Δ45) and differing in the previously reported presence or absence of 57 nucleotides appended to the 3′ end of exon 40 (Table 2). Exon 40 without an extension (40,41,42,43,44,-,46) was expressed in all individuals examined and only one clone of ten isolated from a single individual contained exon 40 with the 57 nucleotide extension (40 nt,41,42,43,44,-,46).

### 3.2 Different Effects of Clevidipine on the Identified Ca_V_1.2 Splice Variants

**Fig. 2:**
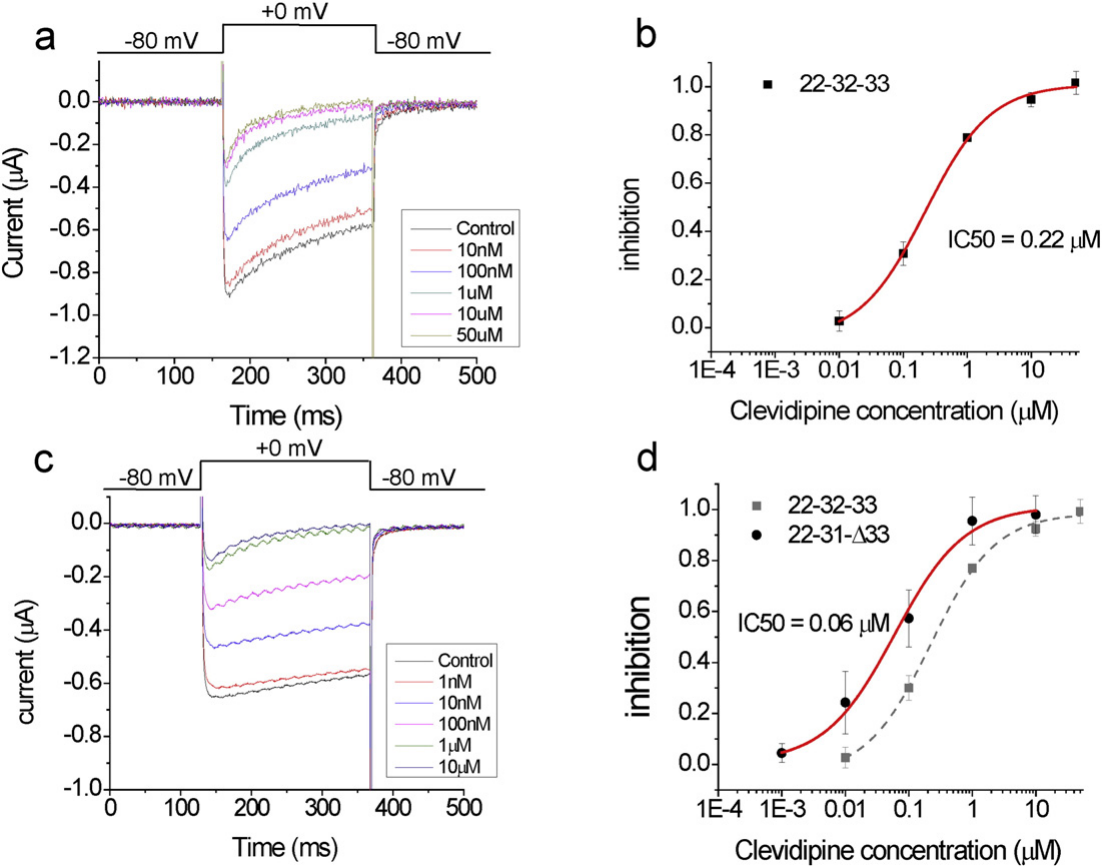
Ca_V_1.2 splice variants have different affinity for clevidipine. (A and C) Currents from A) the 22-32-33 and C) 22-31-Δ33 Ca_V_1.2 splice variants in response to a 0 mV step from a holding voltage of − 80 mV, followed by a tail voltage of − 80 mV, in the presence of the indicated concentration of clevidipine. (B and D) Average dose response (*n* = 4) for B) the 22-32-33 and D) 22-31-Δ33 Ca_V_1.2 splice variants fitted with Hill equation (See Table 3 for parameters).

Because significant *CACNA1C* splice variations in different exons were seen both within individuals and between individuals, cDNAs encoding combinations of these exon variants were constructed, transcribed, and injected in Xenopus oocytes. We tested eight splice variant combinations of *CACNA1C* exons found in human lung tissue (Table 3). All of these eight variants had the same exons in the regions of exons 7–11 and 40–46 [i.e. (7,8,-,9,-,10,-,11), and (40,41,42,43,44,-,46)]. In the regions of exons 20–24 and 30–34, they were variable in exons 21/22, 31/32 and whether exon 33 was present or absent. For example, variant 22–31-Δ33 contains exons (7,8,-,9,-,10,-,11), (20,-,22,23,24), (30,31,-,-,34), and (40,41,42,43,44,-,46). We recorded the currents from these splice variants in response to a 0 mV depolarization from a holding potential of − 80 mV (Fig. 2A). Currents were recorded in 20 mM barium to avoid activating contaminating calcium-activated chloride channels in *Xenopus* oocytes. Extracellular application of clevidipine blocked the currents in a dose dependent manner (Fig. 2A–B). Most of the different splice variants had similar currents and similar responses to clevidipine (Fig. 2B; Table 3). For most of the splice variants, the currents versus clevidipine concentration data could be fitted with a dose response curve with an IC50 around 200 nM. However, the splice variant combination 22-31-Δ33 showed an approximately 4-fold higher affinity (IC50 = 60 nM) for clevidipine compared to the other tested splice variants (Fig. 2C-D; Table 3).

**Fig. 3:**
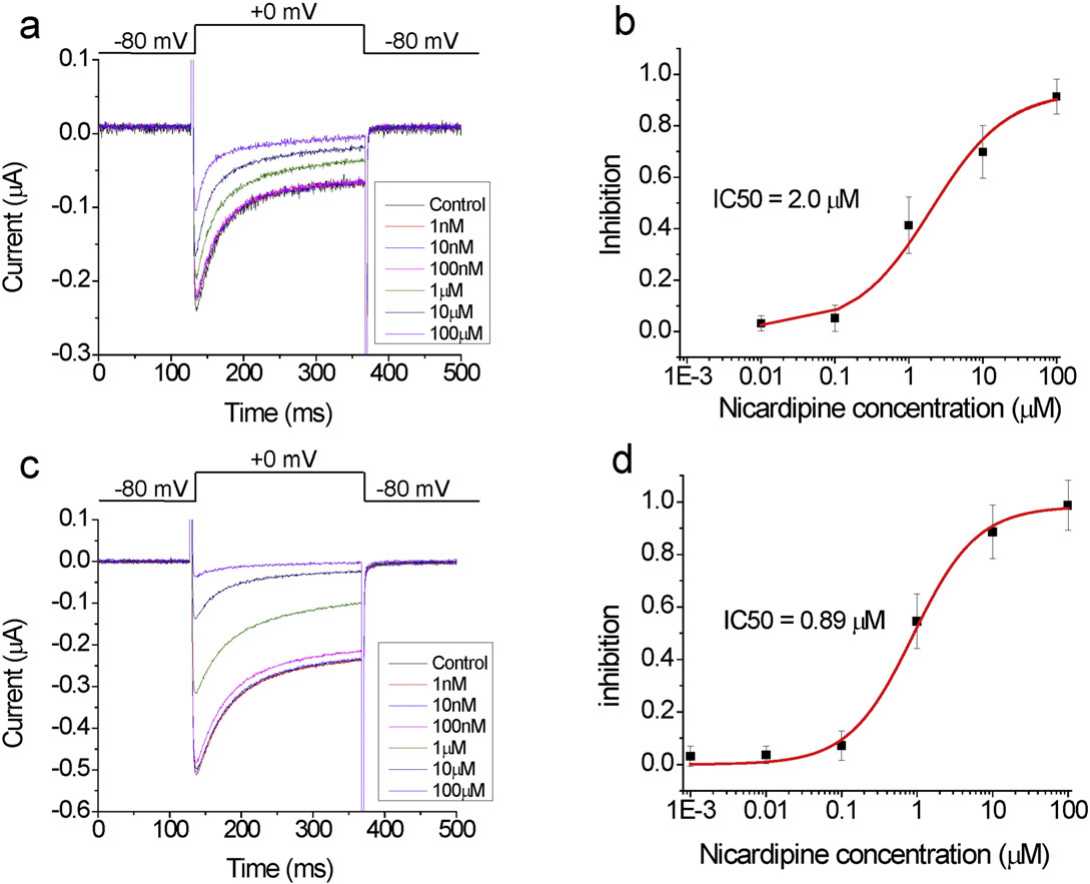
Ca_V_1.2 splice variants have lower affinity for nicardipine. (A and C) Currents from A) the 22-32-33 and C) 22-31-Δ33 Ca_V_1.2 splice variants in response to a 0 mV step from a holding voltage of − 80 mV, followed by a tail voltage of − 80 mV, in the presence of the indicated concentration of nicardipine. (B and D) Average dose response (*n* = 4) for B) the 22-32-33 and D) 22-31-Δ33 Ca_V_1.2 splice variants fitted with Hill equation (see Table 3 for parameters).

We also tested the effect of the DHP nicardipine for the different Ca_V_1.2 splice variants. Nicardipine also blocked current in a dose dependent manner (Fig. 3). However, clevidipine showed an approximately 10-fold higher affinity than nicardipine for the same Ca_V_1.2 splice variants, including the high affinity version (Table 3).

We conclude that Ca_V_1.2 splice variants with different clevidipine affinities are present in lung tissue and these higher affinity variants also show selective higher affinity for one DHP versus another.

### 3.3 Co-expression of Ca_V_1.2 (22-31-Δ33) with Panx1 Boosts the Affinity to Clevidipine

**Fig. 4:**
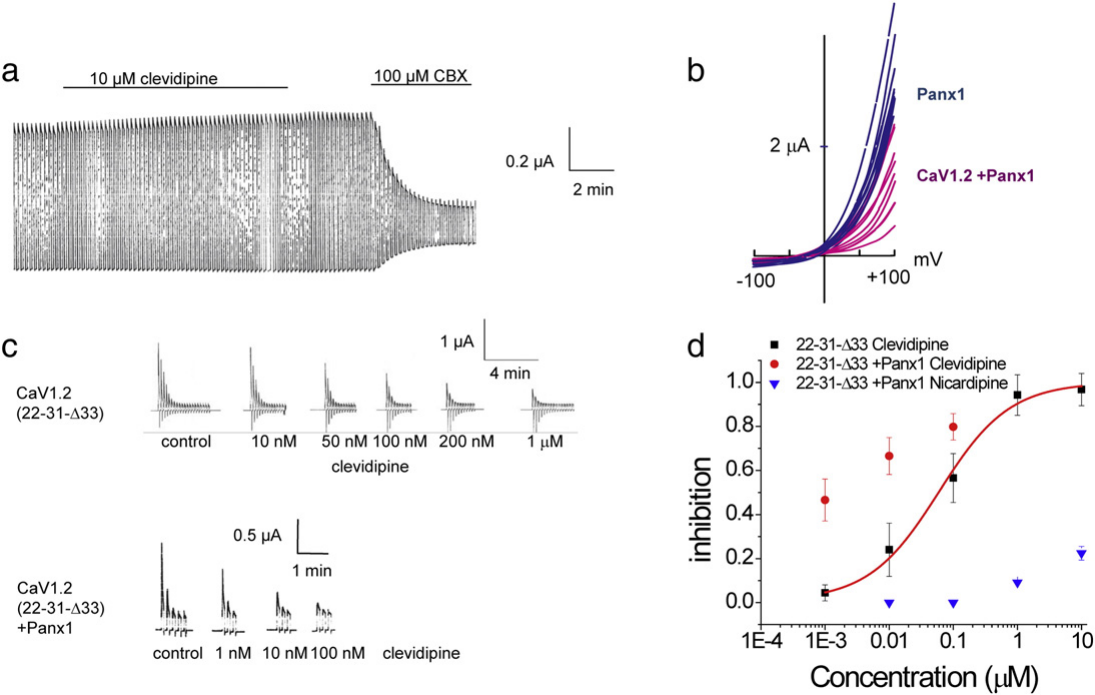
Co-expression of Panx1 with Ca_V_1.2(Δ33) alters sensitivity to clevidipine. A). Clevidipine does not affect membrane currents in oocytes expressing Panx1 channels alone, while the Panx1 blocker carbenoxolone (CBX) attenuates the currents. B). Membrane currents induced by voltage ramps from − 100 mV to + 100 mV in oocytes expressing Panx1 alone (blue traces) and in oocytes co-expressing Panx1 and Ca_V_1.2 (magenta). The exclusively voltage activated Panx1 channel is chloride selective, yielding a strong outward current at positive potentials. The net current in the co = expressing cells is considerably smaller, likely because of the non-selective properties of the Panx1 channels under these experimental conditions. A slight left shift of the reversal potential in the co-expressing cells is consistent with a change in permeability properties. C). Membrane currents induced by voltage steps from − 60 mV to + 60 mV at 0.1 Hz in oocytes expressing Ca_V_1.2 Δ33 alone (top traces) or oocytes co-expressing Ca_V_1.2 Δ33 with Panx1. Records of identical oocytes are shown, where increasing concentrations of clevidipine were applied with 30 minute intervals to allow recovery of the Ca_V_1.2 channels from inactivation. D). Dose-response curves for clevidipine attenuation of currents in oocytes expressing Ca_V_1.2 Δ33 alone (black squares) or oocytes coexpressing Panx1 with Ca_V_1.2 Δ33 (red circles). The co-expressing oocytes were also exposed to Nicardipine (blue triangles).

Pannexin1 channels have been recognized to be involved in the control of smooth muscle contraction. In VSM, pannexin mediates both muscle relaxation and contraction (Billaud et al., 2012, Locovei et al., 2006b). It is therefore conceivable that clevidipine acts on Panx1 as an epiphenomenon and that clevidipine either inhibits Panx1, thereby attenuating α-adrenergic contractions (Billaud et al., 2012), or stimulates Panx1, thereby activating the NO-mediated relaxation. However, when we tested the effects of clevidipine on Panx1 channel currents, we did not observe any direct effect of clevidipine on Panx1 channels expressed in Xenopus oocytes (Fig. 4A).

We also tested the hypothesis that co-expression of Panx1 with Ca_V_1.2 might alter the affinity or behavior of the Ca_V_1.2 in response to clevidipine. I/V curves of oocytes co-expressing Ca_V_1.2 and Panx1 channels exhibit a marked difference between co-expressing cells and cells expressing Panx1 alone (Fig. 4B). The shift in reversal potential and the attenuation of current amplitude are consistent with a Ca_V_1.2-induced switch of the chloride-selective Panx1 channel to the unselective Panx1 (Wang et al., 2014).

The effect of clevidipine on oocytes expressing Ca_V_1.2 (22-32-33) alone or when co-expressed with Panx1 was indistinguishable. However, when Panx1 was co-expressed with the high-affinity splice variant Ca_V_1.2 (22-32-Δ33) a significant left-shift to lower clevidipine concentrations was observed (Fig. 4C–D). No such augmentation for nicardipine affinity was observed (Fig. 4D).

## 4 Discussion

Here we used a reverse translational medicine approach to address a series of clinical observations (Peacock et al., 2014, Kieler-Jensen et al., 2000, Patel et al., 2012) that indicate that the described mechanism of action for clevidipine was incomplete. We hypothesized that molecular variation in Ca_V_1.2 channels exists which confers unique pharmacology upon clevidipine. We report 4 principle findings. 1) Many different Ca_V_1.2 splice variants are present in human lung. 2) Different Ca_V_1.2 splice variants have different affinity for clevidipine. 3) Clevidipine has > 10-fold higher affinity than another similar DHP, nicardipine, to certain Ca_V_1.2 splice variants. 4) Panx1 and Ca_V_1.2 interact, such that Panx1 lowers the IC50 for Ca_V_1.2 splice variants to clevidipine, but not to nicardipine.

The complexity of *CACNA1C* subunit splicing has been previously reported (Hofmann et al., 2014). However, neither the distribution among individuals nor the occurrence of variants in lung tissue has been previously shown. We saw clear differences in expression among individuals, including exons known to affect Ca_V_1.2 functional characteristics. Interestingly, all transcripts from one individual contained either the alternate exon 21 or 22, i.e. no individual expressed both 20,21,-,23,24 and 20,-,22,23,24, whereas variants containing either of the alternate exons 31 and 32 were found in all individuals. Although the functional impact of co-expressing all of the different transcriptional variants is not known, the differential expression could play a role in known individual response variations to DHPs (Cook et al., 1990, Taylor et al., 1985a, Taylor et al., 1985b, Schwieler et al., 1999).

We show that alternative Ca_V_1.2 splice variants have different affinities for clevidipine. One of the tested Ca_V_1.2 splice variants has an IC50 for clevidipine that is approximately 7-fold lower (60 nm) than the reported IC50 for clevidipine in VSM (400 nM) (Nordlander et al., 2004). This pharmacology offers a possible mechanistic explanation underlying the PVR reducing and dyspnea relieving actions of clevidipine, which both occur at lower doses than required for SVR reduction. In addition, we found that clevidipine has > 10-fold higher affinity than nicardipine to specific Ca_V_1.2 splice variants, which could be part of the explanation for the specificity of the dyspnea effect of clevidipine in AHF (Peacock et al., 2014, Cook et al., 1990).

**Fig. 5:**
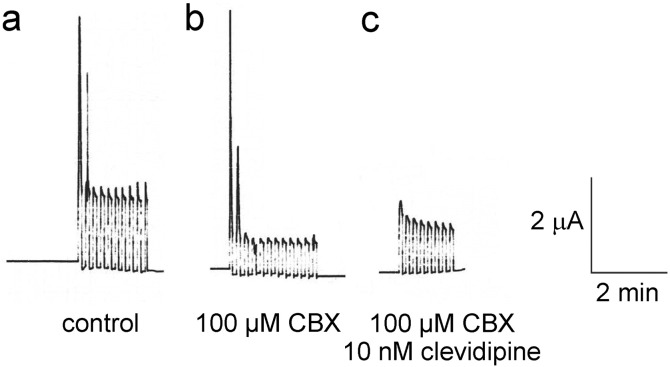
Ionic currents through PNX1 are no necessary for Panx1 to increase the affinity for clevidipine in CaV1.2. Membrane currents observed in oocytes co-expressing Panx1 and CaV1.2 (22,32,Δ33). The pulse protocol was the same as shown in Fig. 4C. After a series of test pulses, oocytes were incubated in Ringer solution supplemented with the Panx1 inhibitor carbenoxolone (CBX), which did not interfere with the large calcium currents at the begin of the pulse series, but attenuated the Panx1 currents remaining after the inactivation of the calcium currents. Subsequent application of clevidipine in the continued presence of CBX resulted in similar inhibition of the calcium currents as in the absence of CBX (cf Fig. 4C).

In addition, we found that Panx1 and Ca_V_1.2 interact, with Panx1 further lowering the IC50 of Ca_V_1.2 for clevidipine (but not for nicardipine). The mechanism for how Panx1 alters the affinity of Ca_V_1.2 for clevidipine is not clear, but ion conduction through the Panx1 channel is not necessary for this effect (Fig. 5). Application of clevidipine in the continued presence of the Panx1 inhibitor carbenoxolone (CBX) resulted in similar clevidipine inhibition of the calcium currents as in the absence of CBX (Fig. 5), indicating that the channel function of Panx1 is not required for the Panx1-induced boost in clevidipine sensitivity of CaV1.2 (22,32,Δ33).

The increased affinity for clevidipine (but not for nicardipine) by the Panx1/Ca_V_1.2 complex could contribute further to the specificity of the dyspnea-relieving effect of clevidipine in AHF (Peacock et al., 2014, Cook et al., 1990). This study shows that Panx1 association alters the affinity of an ion channel to its inhibitor. Although previous studies have shown that Ca_V_1.2 splice variants alter DHP sensitivity, this study shows that different splice variants of Ca_V_1.2 confer differences in the specificity of different DHP.

Further studies are needed to elucidate the mechanism of how Panx1 affect Ca_V_1.2 pharmacology. However, the Panx1/ Ca_V_1.2 interaction observed suggests a possible new paradigm for intracellular calcium regulation: amplification of Ca^2 +^ influx by Ca^2 +^ activation of Panx1, causing Panx1-mediated ATP release. ATP could then act on P2Y receptors to further boost intracellular Ca^2 +^ concentrations via Ca^2 +^ release from intracellular stores. Amplification due to Panx1–mediated secondary ATP release was hitherto only known to boost the response to ligands binding to various receptors, including α-adrenergic agonists, thrombin, angiotensin II, histamine and bradykinin (Dahl, 2015).

A number of questions remain unaddressed. Harvested donor lung likely includes mixed tissue, including lung parenchyma, arterioles, venules, and airway, and, as a result, the tissue location and expression pattern of the splice variants reported here remains unknown. It is possible that the described splice variants are present, but are not physiologically relevant for control of PVR or dyspnea relief. Although this possibility seems unlikely, further studies are clearly needed to define the contribution of these splice variants to both normal physiology and the clinical pathophysiology of AHF. Finally, the extent of any co-localization or co-expression of Panx1 with LTCC in VSM or lung tissue remains unknown.

In summary, we here provide insights into questions raised as a result of clinical experiences: How does clevidipine cause dyspnea relief in hypertensive AHF? What explains the specificity of this effect for clevidipine and not for other DHPs? What underlies the apparent potency differences between clevidipine effects on pulmonary and peripheral circulations? Could clevidipine cause dyspnea relief independent of any BP lowering effects? We here describe new Ca_V_1.2 splice variants in human lung that have increased, selective affinity for clevidipine compared to other DHPs. Panx1 association with this high-affinity Ca_V_1.2 variant further augments its affinity for clevidipine, but not for nicardipine. These observations could explain many of the clinical differences noted above. These experiments refine our understanding of how Ca_V_1.2 alternative splicing produces specific molecular pharmacologic profiles among drugs of the same class. Because these pharmacologic profiles can explain important clinical observations, the possibility now exists to deploy more refined clinical trial designs and endpoints to better examine the role of DHP in the treatment of pulmonary hypertension and acute hypertensive heart failure.

## Author Contributions

GPD, GEC, JAC and HPL designed the study, GPD, GEC and HPL conducted experiments, analyzed the data, and wrote the manuscript, FQ and JW conducted experiments and analyzed the data, ES and JAC wrote parts of and edited the manuscript.

## Figures and Tables

**Table 1:** Primer sequences.

Forward exon7	CCAGCAGAAGATGACCCTTC
Reverse exon11	GACTTGGAGATCCGGTGG
Forward exon20	CACGATCTTCACCAACCTGA
Reverse exon24	TCGCAAGATCTTCACGACAT
Forward exon30	AAATCGCCATGAACATCCTC
Reverse exon34	TTGATGAAGGTCCACAGCAG
Forward exon40	TGAACATGCCTCTGAACAGC
Reverse exon46	CTCCGTGTCATGGTTCATCTT
Reverse exon50	TGTTCCGGTTAACTCCAGGT

**Table 2:** Frequency of Ca_V_1.2 variants.

	Individual lung donors							
A	B	C	D	E	F	G	H
Exons 7–11 variants								
7,8,9,10,11	9/9	9/19	16/18	11/16	11/13	12/17		
7,8,9,9*,10,11	0	7/19	2/18	3/16	0/13	5/17		
7,8,9,10,10*,11	0	1/19	0/18	1/16	0/13	0/17		
7,8,9,9*,10,10*,11	0	1/19	0/18	0/16	0/13	0/17		
7,-,-10,11	*0*	*1*/*19*	0/18	1/16	*2*/*13*	0/17		

Exons 20–24 variants								
20,21,-,23,24	10/10	8/8	10/10	10/10	10/10	9/9	0/8	9/9
20,-,22,23,24	0/10	0/8	0/10	0/10	0/10	0/9	8/8	0/9

Exons 30–34 variants								
30,-,32-6 nt,33,34	1/9	2/9	2/8	0/9	0/9	0/8	0/7	
30,-,32,-,34	3/9	3/9	0/8	3/9	2/9	2/8	1/7	
30,-,32-6 nt,-,34	0/9	0/9	0/8	0/9	0/9	1/8	0/7	
30,-,32,33,34	2/9	3/9	3/8	3/9	5/9	2/8	2/7	
30,31,-,33,34	1/9	1/9	2/8	1/9	1/9	1/8	1/7	
30,31,-,-,34	0/9	0/9	1/8	0/9	1/9	0/8	2/7	
*30,31,32,-,34*	*0*/*9*	*0*/*9*	*0*/*8*	*1*/*9*	*0*/*9*	*0*/*8*	*0*/*7*	
*30,31,32,33,34*	*2*/*9*	*0*/*9*	*0*/*8*	*1*/*9*	*0*/*9*	*0*/*8*	*0*/*7*	
*30,-,-,33,34*	*0*/*9*	*0*/*9*	*0*/*8*	*0*/*9*	*0*/*9*	*2*/*8*	*0*/*7*	
*30,-,-,-,34*	*0*/*9*	*0*/*9*	*0*/*8*	*0*/*9*	*0*/*9*	*0*/*8*	*1*/*7*	

Exons 40–46 variants								
40,41,42,43,44,-,46	9/9	8/8	3/3	9/10	8/8	14/14		
40 + 57 nt,41,42,43,44,-,46	0/9	0/8	0/3	1/9	0/8	0/14		

Italicized entries are not expected to be functional.

**Table 3:** IC50 and Hill coefficient for the different Ca_V_1.2 splice variants. IC50 and Hill coefficient (h) from fits of data as in Fig. 2 with the equation I (concentration) = I(0) ∗ 1 / (1 + (IC50 / [concentration])^h^).

Clones	Drug	IC50 (μM)	h
22-32-33	Clevidipine	0.22	0.85
22-32-33	Nicardipine	2.01	0.85
21-31-33	Clevidipine	0.19	0.82
22-31-delta33	Clevidipine	0.06	0.8
22-31-delta33	Nicardipine	0.885	1.03
22-32-delta33	Clevidipine	0.19	1.1
21-32-delta33	Clevidipine	0.28	1.04
21-31-delta33	Clevidipine	0.16	0.9
